# Examining intersections between violence against women and violence against children: perspectives of adolescents and adults in displaced Colombian communities

**DOI:** 10.1186/s13031-019-0200-6

**Published:** 2019-06-11

**Authors:** Jennifer J. Mootz, Lindsay Stark, Elizabeth Meyer, Khudejha Asghar, Arturo Harker Roa, Alina Potts, Catherine Poulton, Mendy Marsh, Amy Ritterbusch, Cyril Bennouna

**Affiliations:** 1 Department of Psychiatry, Columbia University, New York, NY USA; 2 New York State Psychiatric Institute, New York, USA; 3 Program on Forced Migration and Health, Mailman School of Public Health, Columbia University, New York, NY USA; 4 George Warren Brown School, Washington University in St. Louis, 1 Brookings Dr, St. Louis, MO 63130 USA; 5 Department of Population,Family, and Reproductive Health, Johns Hopkins Bloomberg School of Public Health, Baltimore, USA; 6 School of Government, Universidad de los Andes, Bogotá, Colombia; 7 UNICEF Office of Research-Innocenti, Florence, Italy; 8 UNICEF, 3 United Nations Plaza, New York, NY 10017 USA; 9 The Equality Institute, Melbourne, Australia

**Keywords:** Armed conflict, Domestic violence, Child abuse, Internal displacement, Social norms, Violence against children, Colombia

## Abstract

**Background:**

Research examining the interrelated drivers of household violence against women and violence against children is nascent, particularly in humanitarian settings. Gaps remain in understanding how relocation, displacement and ongoing insecurity affect families and may exacerbate household violence.

**Methods:**

Employing purposive sampling, we used photo elicitation methods to facilitate semi-structured, in-depth interviews with female and male adolescents and adults aged 13–75 (*n* = 73) in two districts in Colombia from May to August of 2017. Participants were displaced and/or residing in neighborhoods characterized by high levels of insecurity from armed groups.

**Results:**

Using inductive thematic analysis and situating the analysis within a feminist socioecological framework, we found several shared drivers of household violence. Intersections among drivers at all socioecological levels occurred among societal gender norms, substance use, attempts to regulate women’s and children’s behavior with violence, and daily stressors associated with numerous community problems. A central theme of relocation was of family compositions that were in continual flux and of family members confronted by economic insecurity and increased access to substances.

**Conclusions:**

Findings suggest interventions that systemically consider families’ struggles with relocation and violence with multifaceted attention to socioecological intersections.

## Background

Over the past decade, women’s and children’s exposure to interpersonal violence has been increasingly recognized as a public health priority [1–3]. This priority is underscored by research demonstrating the harmful effects of violence on women’s and children’s physical and mental health throughout the life course [4–7]. While the forms and effects of violence may differ substantially for girls, boys, women, and men in the aggregate, violence against women (VAW) and violence against children (VAC) both negatively impact gender equity and economic growth at a population level [8–10]. A recent rise in the number of studies examining overlapping risk factors, shared root causes, and consequences of violence on wellbeing reflects increased interest in this topic [11–13].

Household violence—defined as “power and/or control perpetrated by one person in the household, with the intention or effect of causing harm to another person in the household’s physical, sexual, or emotional health or well-being” [14–16]—has intergenerational effects [9]. An analysis of the UN Multi-Country Study on Men and Violence in Asia and the Pacific concluded that childhood trauma, including past victimization, led to a greater likelihood of perpetration or experiences of violence in adulthood across multiple contexts [17–19]. Both boys and girls with maltreatment experiences, including exposure to household violence, are more likely to grow up to perpetrate aggression in adolescence and adulthood [20–25], often in the form of intimate partner violence (IPV).

Colombia is an upper-middle income country that housed the world’s largest number of internally displaced persons in 2016, has experienced decades of civil war between the government and non-state armed groups, and offers a window into the effects of protracted conflict on VAW and VAC [26, 27]. In Colombia, conflict has led to widespread displacement and the proliferation of criminal activity [28], which in turn has contributed to community and family violence [29]. In a nationally representative survey in Colombia, 38% of women and girls aged 15–49 reported being physically or sexually abused by a current or recent intimate partner [30].

Given that humanitarian contexts, in particular, present situations of grave concern for women and children with regards to exposure to violence [31, 32], increasing attention has been paid to developing and implementing responses to address violence in these settings. Even though violence against children and violence against women are intrinsically linked from a life course perspective, approaches to addressing violence against women and violence against children in emergencies have evolved along different trajectories over the years, and have constituted two distinct fields of practice: the ‘gender based violence’ and ‘child protection’ sectors, as they are known within the humanitarian response architecture. While there are important and strategic reasons to separate advocacy and programming for women and children, insufficient attention has been given to opportunities to leverage programming where there are linkages, particularly around intersecting risk factors in the home.

Recently, a structured review consolidating evidence concluded that conflict exposure, alcohol and drug use, income/economic status, mental health/coping strategies, and limited social support were shared drivers of violence for both women and children in the home [33]. While the Rubenstein et al. review provides an important distillation of the current quantitative literature, more nuanced qualitative learning is also needed to understand how these factors may co-occur and how they affect the experience of affected populations. Additionally, qualitative learning may help to suggest more integrative frameworks that may be useful for programmers.

The study outlined in this paper draws on an adapted version of Brofenbrenner’s socio-ecological framework [34]. This framework recognizes that child development is shaped by nested layers of influencing factors. The framework has been adapted and expanded by the public health community [35, 36] and gender-based violence researchers [37] to explain risk factors for interpersonal violence at individual, relational, community and societal levels.

Drawing upon Bronfenbrenner’s socioecological framework, researchers have written about alcohol use, HIV status, the experience of violence in childhood, and psychological mechanisms (e.g., hostility) as individual level factors for violence against women and children [38, 39]. Examples of relational variables consist of decision-making power, relational quality, sibling mistreatment, mother’s attribution of child intentions, and exchanges wherein the victim challenges the perpetrator [37, 40, 41]. Community variables include exposure to armed conflict, infrastructural limitations (such as poor rule of law), migrant status, residential instability, neighborhood structural factors, and community norms [42–45]. Lastly, at the societal level researchers have examined the relationship between patriarchal structural inequities and violence against women and children [39, 46].

### The present study

Our study, funded by the United States Office of Foreign Disaster Assistance (OFDA), aims to address gaps in knowledge around intersections of violence against women and children in humanitarian contexts. While there has been some progress in examining forms of household violence as interrelated, little work has examined the specific and potentially discrepant ways that shared drivers of VAW and VAC operate at various socioecological levels. Additionally, there is limited understanding of how forced migration and displacement relate to VAW and VAC in the home. As most violence against women and children occurs in the context of the home -- even in humanitarian settings [47], the home was chosen as a focal point for this study.

Using qualitative methods to better understand drivers of household violence in a setting characterized by relocation and displacement, the present study was guided by the following two research questions. [1] What are the local drivers and social norms affecting household violence? [2] How does exposure to conflict and/or relocation affect families?

## Methods

### Research setting and participants

The study was conducted with Spanish-speaking adolescents aged 13–17 and adults aged 18–75 in the Cundinamarca and Córdoba departments of Colombia. These two settings, chosen in consultation with Universidad de los Andes and UNICEF Colombia, had different urban/rural distributions but encompassed neighborhoods characterized by high levels of displacement and/or insecurity with an ongoing presence of armed groups. In Cundinamarca, the urban context was a large informal settlement on the outskirts of Bogotá, in which limited access to basic necessities such as running water and public transport exist alongside armed group activity and distrust of local authority figures, such as police, representing the state [48, 49]. Córdoba, located in the north of the country, has been described as the “epicenter of the paramilitary movement” [50], and hosts over 300,000 persons displaced by conflict between military, paramilitary, and local guerrilla forces [51, 52].

### Instruments

We used photo elicitation methods to facilitate open-ended, in-depth individual interviews on topics related to gender norms, household violence, and wellbeing [53–55]. Photo elicitation is a participatory, qualitative method that uses photography as a tool for facilitating discussions on sensitive topics, such as VAW and VAC [54]. Multiple interviews were conducted to build rapport with the same participants over time, and photo-taking was used to encourage participant-initiated conversation on thematic content of sessions. Participants were asked to take photos related to prompts about family relationships (interview 1), family safety and wellbeing (interview 2), changes to family dynamics during times of displacement or insecurity (interview 3), and gender norms (interview 4).

### Data collection

Our community-based partners purposively selected one member per household for photo elicitation interview activities. Community partners were given information on the desired sample, which aimed to recruit equal numbers of men, women, boys, and girls, and approached members of households who were known to have experienced displacement and/or were living in neighborhoods with large known concentrations of displaced households to invite one person to participate based on the specified criteria. Community leaders were encouraged to use their own discretion and judgment in discussing displacement explicitly. Due to stigma about disclosing displacement status and guidance from community partners in urban areas, displacement status was not used as study eligibility criteria; participants were asked about relocation more broadly during interviews, which provided an opportunity to disclose displacement status. The field team included four Colombian researchers from diverse academic backgrounds, a Colombian field coordinator, and two American field oversight staff. Interviewers (three women, one man) were based in Bogotá and completed a two-week training on qualitative research methods, interview tools, ethics and confidentiality, conceptions of violence, and reflexivity. Local referral mechanisms for survivors of violence were identified in both sites and interviewers followed locally approved referral protocols. All data collection procedures were approved by Institutional Review Boards at both Columbia University (AAAR1039) and Universidad de los Andes, and completed in accordance with World Health Organization ethical guidelines for research (2011).

Interviewers obtained consent from adult participants and caregivers and assent from adolescent participants to complete three to four interviews, depending on participant availability, in confidential spaces managed by non-governmental organizations. All consent and interview procedures were completed in Spanish. Consent was also obtained to audio-record interviews, and detailed notes were taken if consent for audio-recording was denied.

To protect safety and confidentiality of participants, the study was presented as a family well-being study to community members. After obtaining consent, interviewers introduced the activity and asked participants to elaborate on topics related to the thematic session. Participants who did not possess photo-taking devices were provided a mobile phone with photo-taking capability to use for the duration of the study. Many participants had a mobile phone with photo-taking capabilities, and opted to use their own phone rather than a phone provided by the study team. Participants were instructed to only use the phones if they felt safe doing so, and encouraged to consider taking photos of inanimate objects or other items that they felt were symbolic of the themes and interactions between family members, as opposed to taking photographs of individuals. Additionally, community partners included organizations that conducted various types of sports and arts activities with adults and youth, and this link with community partners may have helped reduce suspicion about use of phones for photo-taking.

Between May and August 2017, 226 interviews were completed with 73 participants (29 women, 13 men, 15 adolescent girls, and 16 adolescent boys). The average age of adolescents was 15 years (*SD* = 1.5) and adults 44.3 years (*SD* = 13.9). While the numbers of girls and boys in the sample were about equal, 58% of adults in the sample were women.

### Data preparation and analysis

Interviews were transcribed into Spanish and then translated into English, using a professional translation service in Colombia. The subset of interviews that were reviewed as part of this analysis were drawn from 18 women, 13 men, 15 adolescent girls, and 16 adolescent boys to account for oversampling of women in Cundinamarca. Interviews were selected to maximize inclusion of perspectives from different participants and to include an equal number of interviews per photo elicitation session.

We used an inductive thematic approach to analyze data [56]. Eight staff in an academic setting in the U.S. participated in transcript review and codebook development. In accordance with recommendations on including the language of completed interviews in analysis, two staff members with Spanish fluency reviewed 13 transcripts in Spanish, and an additional six staff reviewed the remaining transcripts translated from Spanish to English [57]. Using Dedoose version 8.0.42, the analysis team developed open codes through a random selection and review of an adolescent boy’s transcript and revised the codebook against transcripts from the other subgroups, using different sessions of the photo elicitation interviews. Weekly team meetings included discussions of code definitions and researcher biases through review of transcripts. Interpretive reflections and analytic memos documented during open coding informed development of central themes. Following inductive analysis, we situated the overall themes within the socioecological framework (i.e., individual, relational, community, societal levels).

## Results

The experience of relocating from a rural to an urban environment was commonplace. Respondents highlighted several reasons for relocation, though the most commonly cited scenarios relayed experiences with interpersonal violence—either as respondents had experienced or witnessed it in the community or within the household. Some respondents who had relocated from rural areas, for instance, described relocating because of armed conflict and rebel group activities.

*We lived in an area called [name of community], here on this side. Well, we left. We grew up in the country, and we lived very happily in the country until that moment when, well, the armed groups had already started to invade the country. And then there was an armed confrontation. Eh, guerillas attacked a paramilitary base in that area and there was a lot of killing—that horrible, horrible, horrible, horrible fighting.*

*– Adult Man, Age 60, Tierralta*

However, many respondents (especially women and children) described relocation as a response to experiencing household violence between intimate partners or against children.

*When we were little, my dad, he drank a lot, so he would come home and hit my mom. So, when we were little, we would see that. So, then when my mom made the choice to leave him, for us it was difficult.*

*– Adolescent Girl, Age 17, Soacha*

### Drivers of household violence

Respondents described drivers—factors preceding and likely contributing to VAW and VAC—at all levels of the socioecological model. Table 1 details where the drivers of VAW and VAC situate within the socioecological model, as well as the overlapping and contrasting ways in which these drivers manifested between VAW and VAC. The drivers of substance use and accumulation of daily stressors were associated with relocation experiences.

**Table 1:** Socioecological Drivers of Violence Against Women (VAW) and Violence Against Children (VAC) in the Household

Drivers of Violence	VAW	VAC
Societal Gender Norms (S)	Expectations that men dominate female partners and use aggression to maintain and display dominance	Expectations that adults (both men and women) dominate children
Substance Use (I/C/S)^a^	Male battering of female intimate partner when inebriated	Battering of children (usually by man) when inebriated; beating as a form of punishment for child substance use; parental inebriation associated with failure to protect girls from extended family members’ perpetration of sexual abuse
Accumulation of Daily Stressors (I/C)^a^	Intimate partner and economic stressors (e.g., management of finances and unemployment); unwillingness to share resources; intimate partner disagreement over resources	Management of work and parenting responsibilities compounded by economic stressors; parents batter children while trying to manage routines; lack of food due to displacement heightens child crying and perceived misbehaving
Behavior Regulation (R/S)	Control of intimate partner’s sexual autonomy and access to activities outside the home; reproductive coercion; perceived deviation from female gender roles (e.g., meal preparation)	Punishment for substance use, disobedience, “talking back”, poor school attendance or performance, and missed curfews; perceived lack of contribution to household tasks; hierarchical familial practices of discipline; children (usually boys) attempt to regulate violent behavior of caregivers

*Note. I* individual driver, *R* relational driver, *C* community driver, *S* societal driver

^a^= also related to relocation experience

#### Societal level gender norms

Situated at the outer level of the socioecological model, societal gender norms markedly shaped relational and individual behavior. While respondents did not describe relocation as having directly influenced gender norms, we provide a brief analysis of these norms due to the fact that they were frequently connected to VAW and VAC, offering a contextual backdrop for relocation and resettlement experiences. First, the experience of household violence reflected inequitable gender and age-related power dynamics: women and children were more likely to experience household conflict or violence than men, according to many respondents.

*I’ve seen, in my home we had that case with my dad, the men always try to rule the women, there’s abuse, hitting, or verbal humiliation. They say “no, you’re a women, you’re good for nothing”.*

*–Adolescent Boy, Age 17, Soacha*

The societal gender norms described tended to be associated with attempts to regulate behavior and expectations for male dominance and aggression, especially for male violence against female intimate partners. Adult-perpetrated VAC as a form of behavior regulation also manifested, although perpetrators of VAC tended to be both men and women in examples. Adolescents, especially boys, additionally attempted to use violence to regulate parents’ undesirable behaviors.

*And plus, my dad did use violence, because my dad hit my mom pretty hard for a while, because he saw that his brothers were telling him, “You have to get her in line. You can’t let her talk back to you, you have to…” That is what my mom told me, right? I mean, my dad was a good boyfriend until he went drinking, for example, with his friends, or with his brothers, or the family. They’d tell him, “You have to give it to your wife every now and then. I mean, give it, hit her like to keep her subjugated, in line,” like they said, so my dad would hit us hard.*

*– Adult Woman, Age 33, Soacha*

For women and girls, behavior regulation additionally occurred through the restriction of sexual autonomy, as well as freedom to leave a relationship. As shared by one participant,

*He starts hitting her so she gives him children, or if not he’ll look for another [woman] and try with her like that.*

*– Adolescent Girl, Age 14, Tierralta.*

Another woman shared,

*And that man made a house for her and everything for that girl. And one day he had her killed because she didn’t want to be with him anymore.*

*– Adult Woman, Age 46, Soacha*

VAW and VAC were repeatedly found in connection to the division of and expectations associated with household responsibilities. With regards to VAW, violence stemmed from men perceiving women not to perform expected gendered responsibilities, such as meal preparation. An adolescent girl, age 14, from Tierralta shared,

*The woman has to have food ready for him, and if she doesn’t have it, he beats her.*

A perceived lack of contribution to household responsibilities also drove VAC. In these instances, a range of adult caregivers, including mothers, fathers, and extended family members, perpetrated violence against children. An adolescent boy, age 14, from Tierralta shared,

*Because, I mean, because sometimes I don’t listen to her. I mean I don’t do the errands or that she tells me to, I leave and I go somewhere else and she scolds me, sometimes she hits me.*

For children, violence as a form of punishment occurred in response to disobedience, poor school attendance or performance, substance use, and missed curfews. Some respondents normalized VAC as a parenting technique. For example, a woman, age 35, from Tierralta shared,

*One day I hit him…you have to, because sometimes they’re lost/clueless.*

The quotation below shows that these parenting practices were often intergenerationally learned parenting techniques to regulate behavior.

*Because she would get so ma— well, she says that, that’s how they used to teach you before, and she like learned that and copied it, to like teach me, to teach me, because that’s how they used to teach you, before. And she had, I mean, she had that like habit from when they used to hit her, and so she thought, well, I’ll hit my children, too, like they taught me to.*

*– Adolescent Girl, Age 15, Soacha*

Adolescents also reported attempting to regulate and interrupt male perpetrators’ violent behavior against mothers, which resulted in VAC. Boys were often the targets of VAC in this context.

*Respondent (R): …He returned after some time to see if his father had changed, but he hadn’t. He was worse. He would come home drunk, when he wanted to hit the mother and things like that. Interviewer (I): Then he would try to defend the mother.*

*R: Since there are children who don’t want anything to happen to their mother. And he would do that, but he would also get hit.*

*– Adolescent Girl, Age 15, Tierralta*

### Relocation: interactions among families, communities, and individuals

**Fig. 1:**
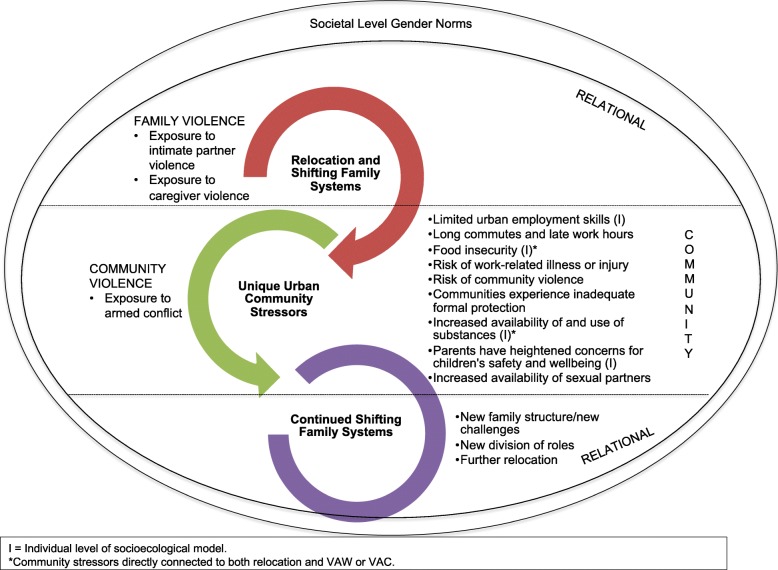
Socioecological Conceptual Figure of Relocation Challenges in Colombia

Respondents rarely directly connected the experience of relocation or exposure to conflict with increased household violence, despite relocation reportedly having negatively affected families in several ways. When respondents identified relocation as directly related to VAW and VAC, their narratives revealed pivotal interactions among families, urban community systems, and individual-level problems (see Fig. 1). Linkages between relocation and drivers of VAW and VAC were more often made indirectly, through discussions of heightened exposure to insecurity in the community, increased availability and use of substances and exposure to and accumulation of daily stressors that negatively affected families’ and individuals’ functioning, as described below.

#### Shifting family systems

First, at the relational level, there emerged the theme of instability through the continual shifting of family structures and composition. While not absolute, shifting family systems often depicted a core family composition of women and their children. As one woman shared,

*Some [families] that I know the woman is single because they killed the husband. They are displaced, and the woman makes the decisions.*

*–Adult Woman, Age 47, Tierralta.*

Another adolescent male shared:

*I had to receive [my aunt] because she had no place to go, and she came with two little children and, because her mom and because the cousin that I told you I have here, there’s a daughter of her, and she has two other little ones. So, there are four little children arriving to the house at six in the morning and with another cousin that goes there, her niece and another little boy, like five… it’s a lot of disorder.*

*–Adolescent Boy, Age 19, Soacha*

In addition to losing husbands to conflict, participants described women divorcing or separating from abusive male partners and sometimes later partnering with other men:

*Because we were used to seeing them [mom and dad] both, so then when they split up, well my nineteen-year-old sister, she left with him, my brother. Well, he started to smoke and was sent to a boarding school, and so we well, stayed with my mom and that’s when she found my stepfather.*

* – Adolescent Girl, Age 17, Soacha*

The shifting of family structures was a dynamic process that interacted with the host community environment. Family compositions changed when relocating and continued to shift due to stressors faced in the host community. The need for income generation as well as exposure to community violence were associated with continued instability and separation of family members, as illustrated in this narrative:

*So they began to give him work, and he was working, and he began to hang out with all those guys, the ‘patos’ [bus driver’s helper]. Some of them are annoying, some of them bother so they say some of those guys were going to kill him because once he saw a thing that they were going to assault a bus in which he was working as a ‘pato’ and they killed the driver. He saw everything. He saw who was the one who killed the driver so they were going to kill him because of that, because he could talk, so that’s why they [his family] took him away from here.*

* – Adolescent Boy, Age 17, Soacha*

#### Urban Community stressors

At the community level, almost all participants described their communities as being unsafe, unprotected, and as posing economic challenges for families. In the following narrative, an adult man draws a distinction between the new urban setting and a former rural setting, perceiving the urban community as more dangerous.

*There are not so many conflicts [in our former home]. Well, there are armed issues, the red zone, but you could live in peace, you can talk, look, you live in peace. But here you don’t. There are many dangers.*

*– Adult Man, Age 34, Soacha*

Perceptions of and experiences with (individual level) community violence affected caregiving practices between adult caregivers and children (relational level). Women especially expressed concern about the safety of their children and correspondingly restricted children’s movement outside the home. Lack of community safety also limited leisure opportunities that were outside the home for youth.

*Now I feel overwhelmed by the level of safety in the neighborhood, mostly because of the safety of my children. Because sometimes when you’re lying down and you don’t know if they’re gonna throw a bomb at you or who knows where. So if my kids go out into the street, and then always they always take ages, so I’m there like “Ah, where are you? What did you do? Where and what did you do? Or maybe you’ve done something. What if something’s happened to you?” You’re there like with that, that silence without them. And your heart is just going. You live through it every day.*

*–Adult Woman, Age 35, Soacha*

A core component of heightened familial stress was the intensification of economic adversity when relocating from rural to urban environments in part because of a lack of labor skills and qualifications (individual level) demanded in an urban environment and also due to relative difficulties of securing food in the respective settings. Occasionally, respondents connected economic adversity, in particular food insecurity (individual level), in urban environments to violence and friction in the home between partners and caregivers/children (relational level).

*Yes I have already suffered [after relocation], as my daughter was never taught to endure hunger. When we took her, she was hungry and I did not have food to give her, she would get angry. Then she would make me angry, and I would beat her… That's why I’m saying this—it [living in the rural area] was content. Because in the mountainside you can fish, you kill fish, kill bushmeat. All the children were happy…*

*– Adult Man, Age 58, Tierralta*

*…I live with a lot of stress because of the pace of my life, and because of my financial difficulties and because my, yes over many things, eh, you get stressed. I got stress a lot, a lot, a lot… So that day I don’t know for some reason I got up late, got up in a bad mood, I don’t know, and oh and went and clashed so horribly with my kids, horrible, horrible, yelled at them, punched [name of daughter] over here. She didn’t have breakfast.*

*– Adult Woman, Age 40, Soacha*

Urban employment exacerbated family vulnerability through more indirect pathways as well. For example, there were reports of exposure to community violence because of employment hours (e.g., working late hours) or location (e.g., traveling far from home). These problems in the community in turn produced economic difficulties for the family.

Finding and affording childcare presented as another notable adverse experience following relocation. A few respondents offered examples of leaving children with extended family members, representing another way in which the composition of families shifted during the relocation process.

*The roles changed a lot then because, firstly, I changed husbands, from my children’s father. That was a hard loss that I still haven’t gotten over. I don’t think I’ll ever get over that. Then I was left as a mother and father to my kids. I worked outside the home, and I had to work and come home to see my kids. I used to pay people to look after them for me. I’d pick them up and then the next day carry on the same all over again. And for that same reason, it was so hard for me.*

* – Adult Woman, Age 35, Soacha*

Substance use (individual level) was a frequently referenced driver of household violence, and participants provided descriptions of the ways in which substance use drove violence in the household. Most often, participants described alcohol use as a precursor to male-perpetrated VAW and VAC in the home and often connected substance use to narratives of male dominance and female subordination.

*Yes, many times men are always trying to mistreat women when they are drunk or when they are on drugs… Because when he gets drunk, he begins to say that women are easy, that if he wants any woman he would rape them or whatever he wants, because he is a very bad person when he gets in that, when he is with alcohol or in drugs.*

*– Adult Man, Age 19, Soacha*

The same participant noted that instances of alcohol use and household violence were not limited to VAW perpetrated by intimate partners, but also by fathers against children and adolescents.

*In a safe home, alcohol can affect many things because it can break the bonds, the family, over all the problems they can have. In one day, they can even be hurting the woman they have or the children because of the alcohol problem. They can, eh, do a lot of bad things.*

*– Adult Man, Age 19, Soacha*

To a lesser degree, substance use by children also appeared to drive conflicts in the home with caregivers. For example, adolescents’ substance use was implicated as a reason for parental beating as another manifestation of behavior regulation. In other instances, drug or alcohol use by children created conflict with parents either through parental disapproval or as a result of substance-induced aggression.

Urban communities additionally presented more opportunities to obtain and engage in substance use, a frequently referenced driver of VAW and VAC. Substance use in turn was described as being associated with increased fighting and problems in the community.

*But now there aren’t very many conflicts in the countryside because people say there is peace. There’re a lot of soldiers everywhere, so there isn’t very much conflict. But here in the city, we see a lot of conflict because people want to fight all the time. For example, there’re a lot of people who work all the week to drink on Saturday and Sunday. And in those days, unexpected things can happen. For example, there may be problems, or another person who doesn’t bear what the other person is saying to him, and they can fight.*

*– Adult Man, Age 19, Soacha*

## Discussion

To our knowledge, this is the first study to examine household violence—including VAW and VAC from perspectives of both Colombian adolescents and adults in communities with high levels of displacement and ongoing insecurity. Findings illuminated intersections of drivers and gender norms of VAW and VAC across the four levels of the socioecological framework.

Numerous participants in our sample had experienced relocation, a main driver of which was exposure to interpersonal violence at the relational (i.e., household) or community levels (i.e., armed conflict). Data revealed a pattern of shifting family structures and compositions during the process of relocation, with continued shifts after relocation due to community stressors, economic insecurity and exposure to violence. This finding highlights the contribution of household violence as both a result of and an additional driver of displacement for families already vulnerable in contexts affected by armed conflict. Research in low-income countries and humanitarian settings is needed to better understand familial and community variables’ intersecting effects on wellbeing and cycles of violence.

Urban communities presented more availability of and opportunity to abuse substances for relocated families. At the individual level, male alcohol consumption interacted with societal expectations of male aggression against intimate partners and children and was identified as a major driver of VAW and VAC. Substance use has a well-established association with exposure to and perpetration of interpersonal violence [58, 59]. World Health Organization guidelines have addressed gaps in service within humanitarian settings, providing recommendations for the assessment and treatment of substance use and mental health conditions in emergency settings [60]. However, a systematic review on alcohol use among conflict-affected populations in low-and middle-income countries found no studies on intervention effectiveness [61]. While community level interventions are often effective for large segments of the population, specialized behavioral interventions may also be needed for some individuals [62]. Within conflict-afflicted settings, interventions have rarely addressed harmful substance use [63].

Our results highlight the importance of broadening violence-prevention interventions to account for intersecting influences at all levels of the socioecological model, attending especially to intersections between patriarchal gender roles and other local drivers. Multi-pronged interventions that focus on intersecting forms of violence, including polyvictimization, and target families experiencing insecurities related to income, housing, and political conflict, may have more success in reducing violence in the household [64]. While participants connected social norms to VAW and VAC, they did not directly connect relocation and gendered social norms. Yet, evidence continues to amass demonstrating that exposure to general conflict violence significantly increases the risk of intimate-partner abuse, for example [47, 65].

Social norms interventions to reduce VAC and VAW aim to challenge gender norms, increase advocacy efforts, engage in consciousness-raising actions, involve bystanders and stakeholders, and provide increased access to medical and social services [66, 67]. While social norm change efforts are an increasingly popular intervention approach, they have been primarily implemented and evaluated in high income countries [68]. Cislaghi and Heise (2018) have recently offered a framework that uses an adapted ecological model for using social norms theory to inform health-related interventions in low-income settings. They recommend examining how factors at individual (e.g., knowledge and values), social (e.g., social network), material (e.g., resources), and institutional (e.g., laws and policy) levels affect health outcomes. Our findings suggest certain types of interventions that could be usefully implemented and tested in humanitarian and low-resource settings. For example, multilevel parenting and social norms change interventions, such as the *REAL Fathers Initiative* [69], *One Man Can* [70], *Parenting for Respectability* [71], and *SASA!* [72], have demonstrated reductions in both VAC and IPV in low-resource settings and could usefully be adapted and tested in humanitarian settings. Our data suggest that parenting and other family functioning interventions should be integrated with multi-pronged programs that account for the continual shifting of family structures and economic stressors of unemployment, dangerous employment, and food insecurity during relocation processes. Interventions with structural-level components (e.g., enhancing employment opportunities) may in fact have the greatest impact [73]. While VAC and VAW programming has traditionally been addressed in silos in emergency response settings, our findings highlight the opportunity for collaboration and integrated programming that addresses shared drivers.

While there are several strengths to this study, the findings should be interpreted with consideration of some limitations. Procedural limitations included variation in number and duration of interview sessions to match participants’ availability. This flexibility allowed for greater participation but may have impacted analysis. To address the limitation of oversampling, a subset of interviews was selected from each participant demographic and analyzed until saturation, which risks overlooking certain data points or qualitative outliers as well as jeopardizing participants’ time and effort. Additionally, limited demographic information was collected for this sample in order to protect participant confidentiality, potentially impeding our ability to draw conclusions delineated by more specified demographic data. Relatedly, given the sample size and design of this study, the hypothesized relationships linking personal and household risks with displacement remain untested. Additional research could usefully help elucidate whether links between the drivers identified in this thematic analysis are causal or merely co-occurring.

## Conclusion

This study found that drivers of household violence—violence against women and violence against children—manifested at all levels of the socioecological framework and routinely intersected with patriarchal gender norms. The processes of relocation and displacement led to new vulnerabilities and continued instability in family structures, particularly through urban community stressors including safety, economic and food insecurity, and increased access to substances. Collectively, these findings support interventions that systemically consider families’ struggles with relocation and violence, with multifaceted attention to socioecological intersections.
